# An International Perspective: The Role of Transportation in Supporting Community Mobility Needs of Older Adults

**DOI:** 10.1093/geroni/igaa057.2334

**Published:** 2020-12-16

**Authors:** Anne Dickerson, Brenda Vrkljan

## Abstract

This international symposium brings together leading scholars in occupational therapy research whose shared aim is to support community mobility in older adulthood. in this session, five groups of researchers will share their collective and individual research outcomes supporting continued community mobility of older adults, especially when driving is no longer an option. The first presentation will be their collective international, cross sectional study of 247 older adults from seven countries. This study compared community destinations, methods of transportation and factors that influence community mobility. Each presentation that follows will highlight unique studies with the overarching goal of improving mobility options for older adults to “age in place of choice.” Our discussant will summarize the potential for expanding this research by engaging the audience through interactive questions, as to where local and global opportunities for innovations that support community mobility will be raised.

